# Supplementary material for: Multitarget Action of Xanthones from Garcinia mangostana against α-Amylase, α-Glucosidase and Pancreatic Lipase
Source: Molecules. 2022 May 20;27(10):3283. doi: 10.3390/molecules27103283 (PMC9144329; doi:10.3390/molecules27103283)
Supplement: Supplementary file 1 [file molecules-27-03283-s001.zip › molecules-1714107-supplementary.pdf]

Supplementary materials

Multitarget action of xanthones from *Garcinia mangostana* against α-amylase, α-glucosidase and pancreatic lipase

Juan Cardozo-Muñoz[1], Luis E. Cuca-Suárez[1], Juliet A. Prieto-Rodríguez[2], Fabian Lopez-Vallejo[1], Oscar J. Patiño-Ladino[1,*]

[1]Departamento de Química, Facultad de Ciencias, Universidad Nacional de Colombia, Sede Bogotá, Bogotá, 111321, Colombia; jcardozom@unal.edu.co (J.C.M.); lecucas@unal.edu.co (L.E.C.S.); fhlopezv@unal.edu.co (F.H.L.V.)
[2]Departamento de Química, Facultad de Ciencias, Pontificia Universidad Javeriana, Bogotá 110231, Colombia; juliet.prieto@javeriana.edu.co (J.A.P.R.)
*Correspondence: ojpatinol@unal.edu.co; Tel: +57-1-3165000 (ext. 14485)

Table of contents:

1. Isolation Compound

Scheme S1. Isolation scheme of compounds **1** to **5** from *G. mangostana.*

2. NMR spectra from phytochemistry isolation and synthetics compounds.

Figures S1. ^{1}H-NMR spectra of 9-hydroxycalabaxanthone (**1**).

Figures S2. APT spectra of 9-hydroxycalabaxanthone (**1**).

Figures S3. ^{1}H-NMR spectra of 8-deoxygartanin (**2**).

Figures S4. APT spectra of 8-deoxygartanin (**2**).

Figures S5. ^{1}H-NMR spectra of gartanin (**3**).

Figures S6. APT spectra of gartanin (**3**).

Figures S7. ^{1}H-NMR spectra of α-mangostin (**4**).

Figures S8. APT spectra of α-mangostin (**4**).

Figures S9. ^{1}H-NMR spectra of γ-mangostin (**5**).

Figures S10. APT spectra of γ-mangostin (**5**).

Figures S11. ^{1}H-NMR spectra of fuscaxanthone C (**6**).

Figures S12. APT spectra of fuscaxanthone C (**6**).

Figures S13. ^{1}H-NMR spectra of 3-isomangostin (**7**).

Figures S14. APT spectra of 3-isomangostin (**7**).

Figures S15. ^{1}H-NMR spectra of of BR-xanthone-A (**8**).

Figures S16. APT spectra of BR-xanthone-A (**8**).

Figures S17. ^{1}H-NMR spectra of tetrahydro-α-mangostin (**9**).

Figures S18. APT spectra of tetrahydro-α-mangostin (**9**).

Figures S19. ^{1}H-NMR spectra of 3,6-di-pentoxy-α-mangostin (**10**).

Figures S20. APT spectra of 3,6-di-pentoxy-α-mangostin (**10)**.

Figures S21. ^{1}H-NMR spectra of 3,6-di-methoxy-4-methyl-α-mangostin (**11**).

Figures S22. APT spectra of 3,6-di-methoxy-4-methyl-α-mangostin (**11**).

3. Molecular docking studies.

Table S1. Docking Scoring energies found for compounds **1** to **11** against each enzyme (PL, AA and AG).

Figure S23. Molecular docking interaction found for compounds **1** to **11** against PL enzyme.

Figure S24. Molecular docking interaction found for compounds **1** to **11** against AA enzyme.

Figure S25. Molecular docking interaction found for compounds **1** to **11** against AG enzyme.

4. Kinetic study.

Figure S26. Kinetic found for compounds **1** to **11** against PL enzyme.

Figure S27. Kinetic found for compounds **2** to **9** against AA enzyme.

Figure S28. Kinetic found for compounds **1** to **9** against AG enzyme.

1. Isolation Compound.

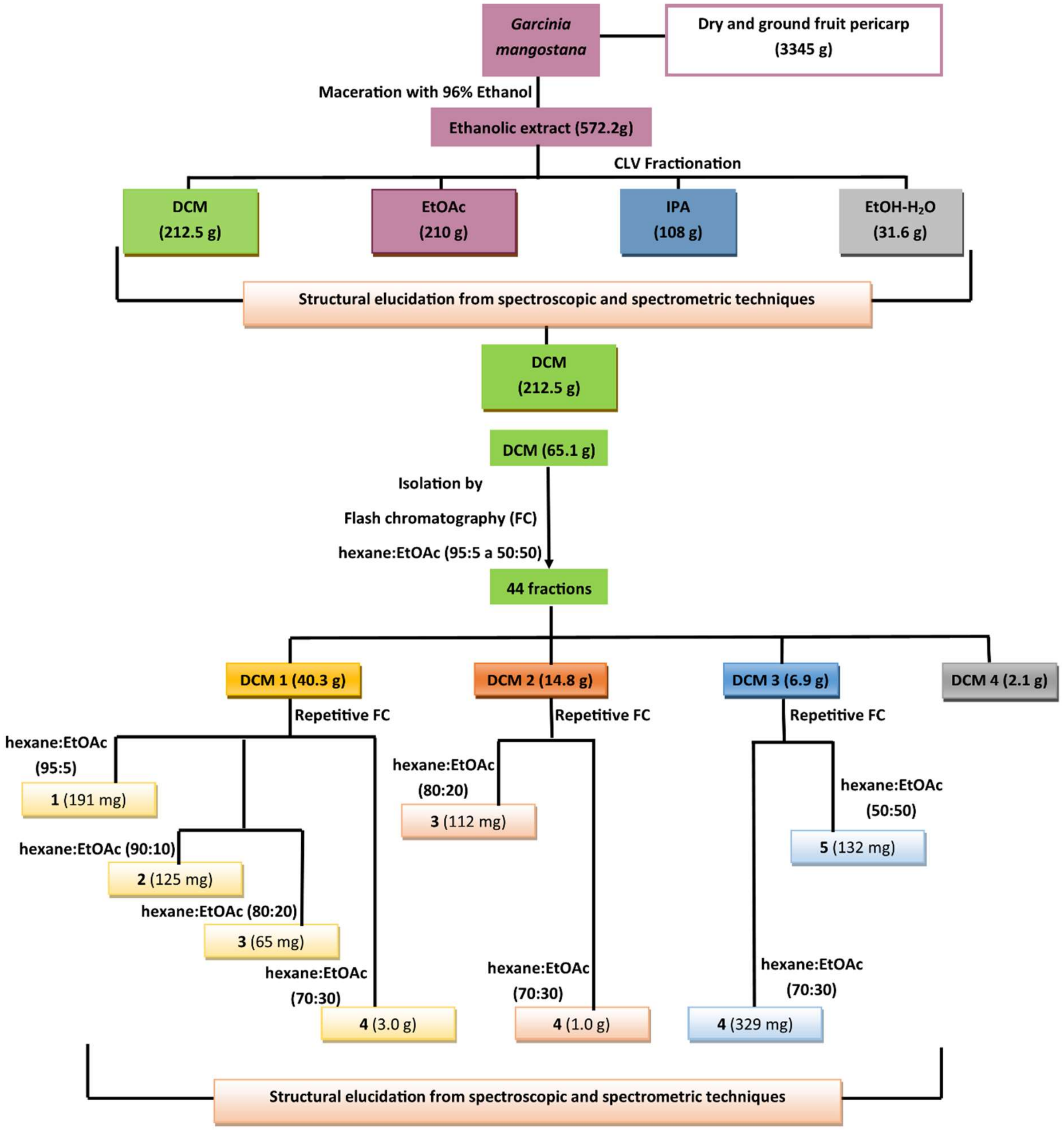

Scheme S1. Isolation scheme of compounds **1** to **5** from *G. mangostana.*

2. NMR spectra from phytochemistry isolation and synthetics compounds.

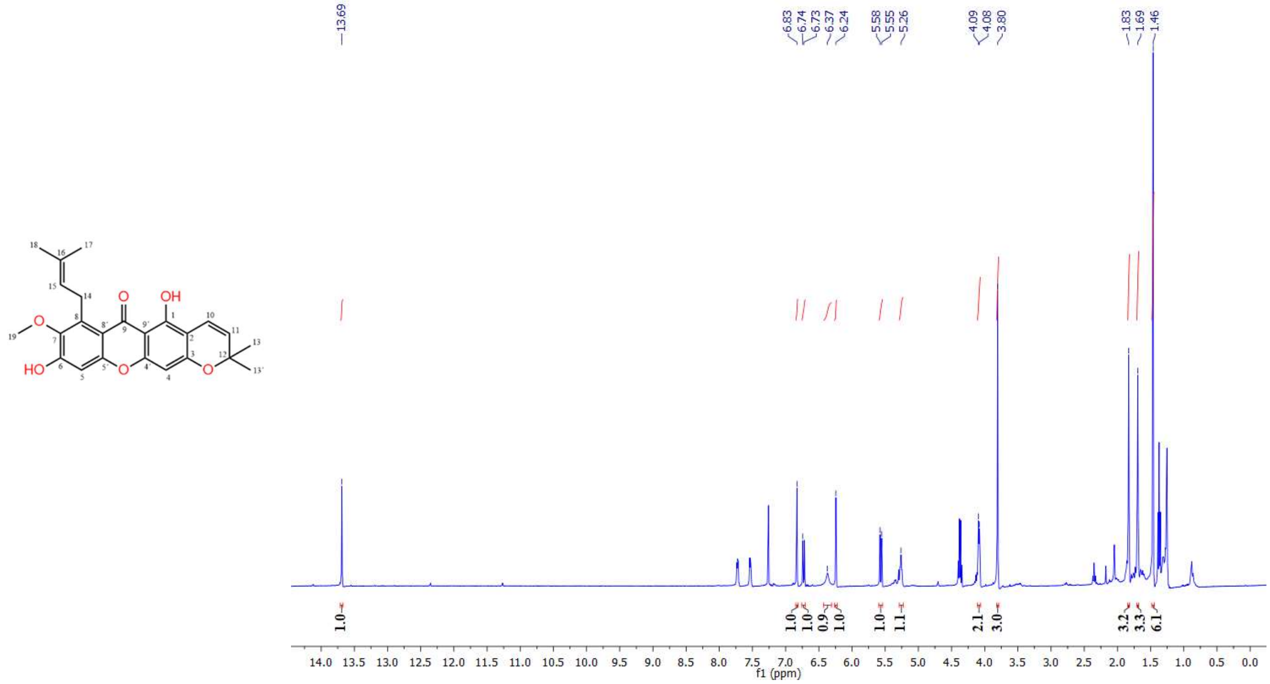

Figures S1. ^{1}H -NMR spectra of 9-hydroxycalabaxanthone (**1**).

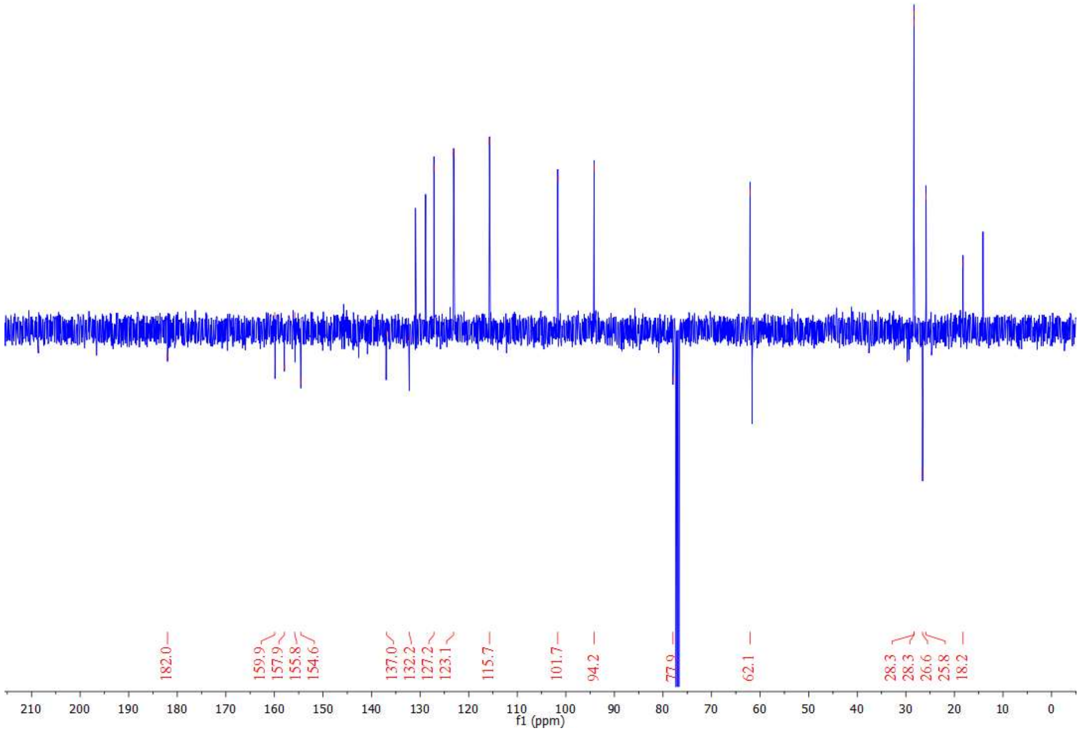

Figures S2. APT spectra of 9-hydroxycalabaxanthone (**1**).

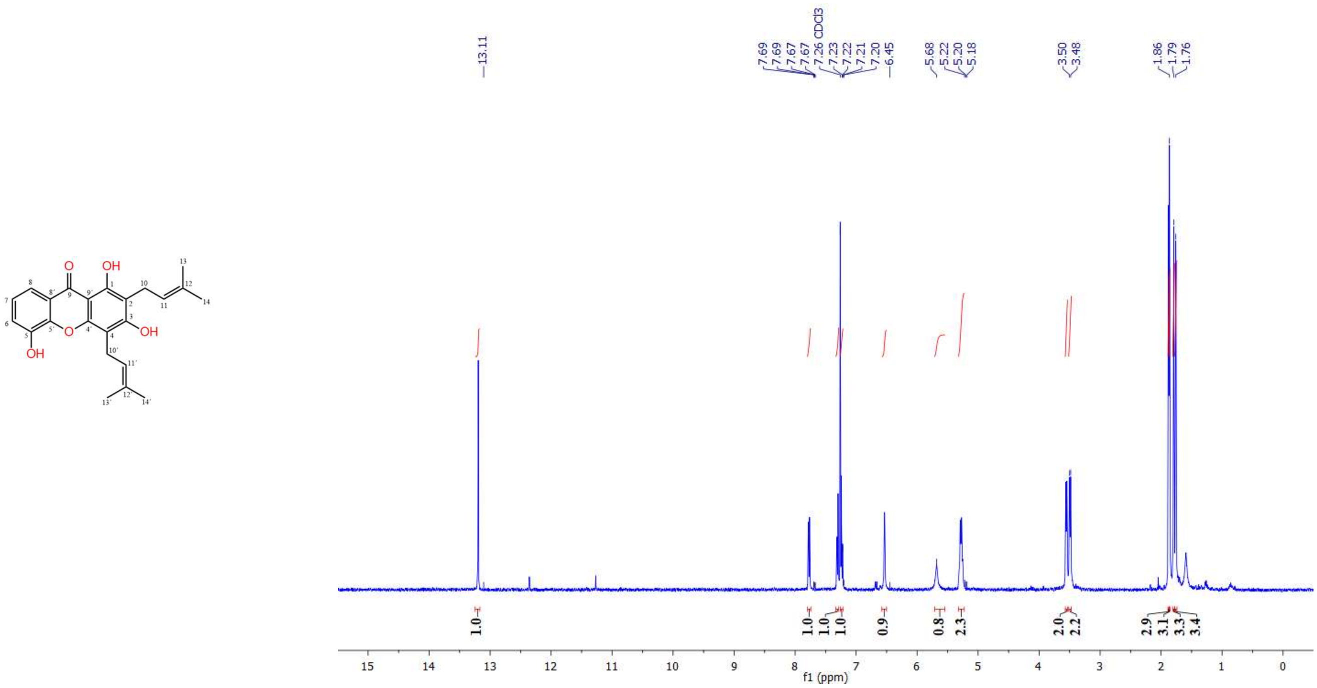

Figures S3. ^{1}H -NMR spectra of 8-deoxygartanin (**2**).

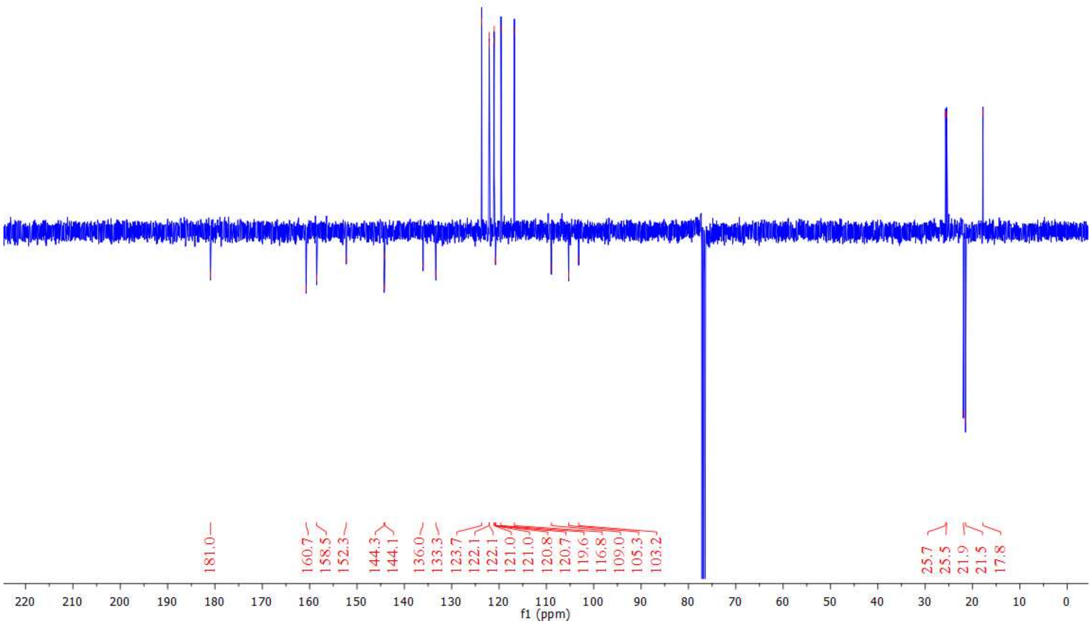

Figures S4. APT spectra of 8-deoxygartanin (**2**).

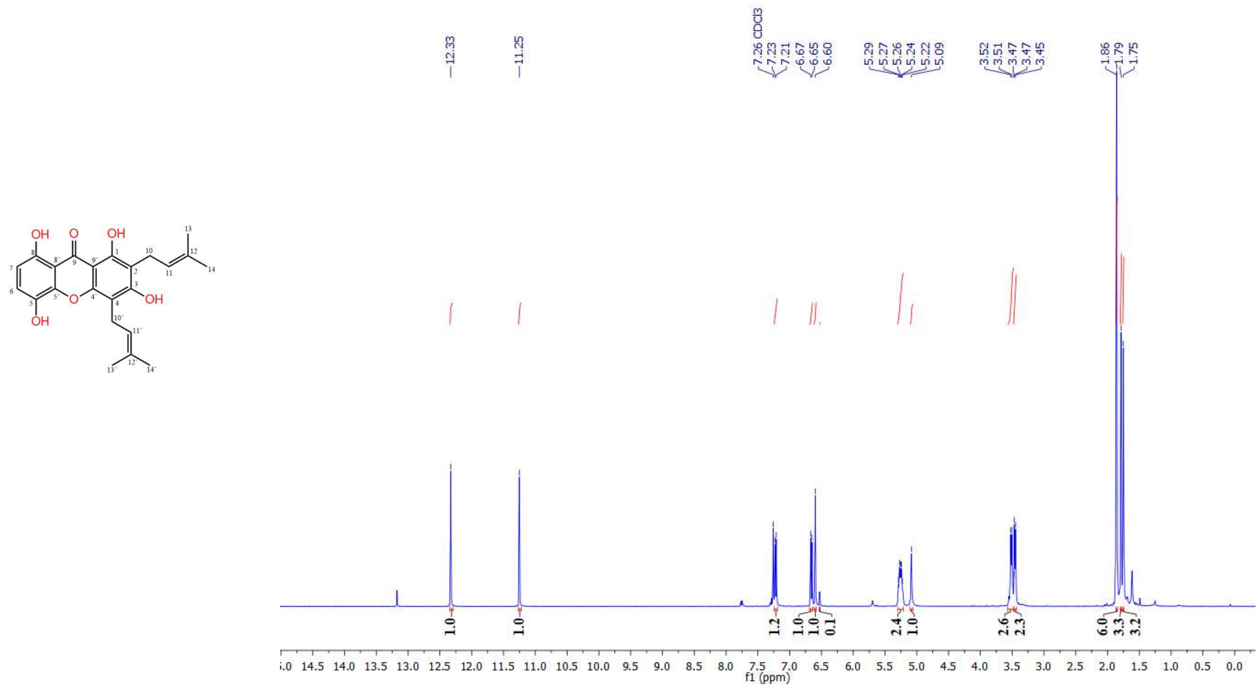

Figures S5. ^{1}H -NMR spectra of gartanin (**3**).

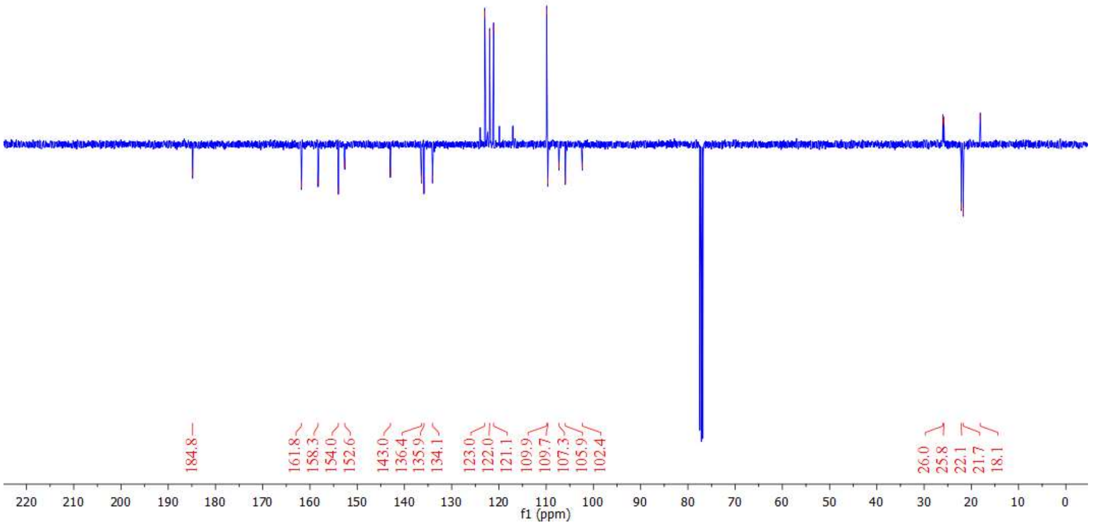

Figures S6. APT spectra of gartanin (**3**).

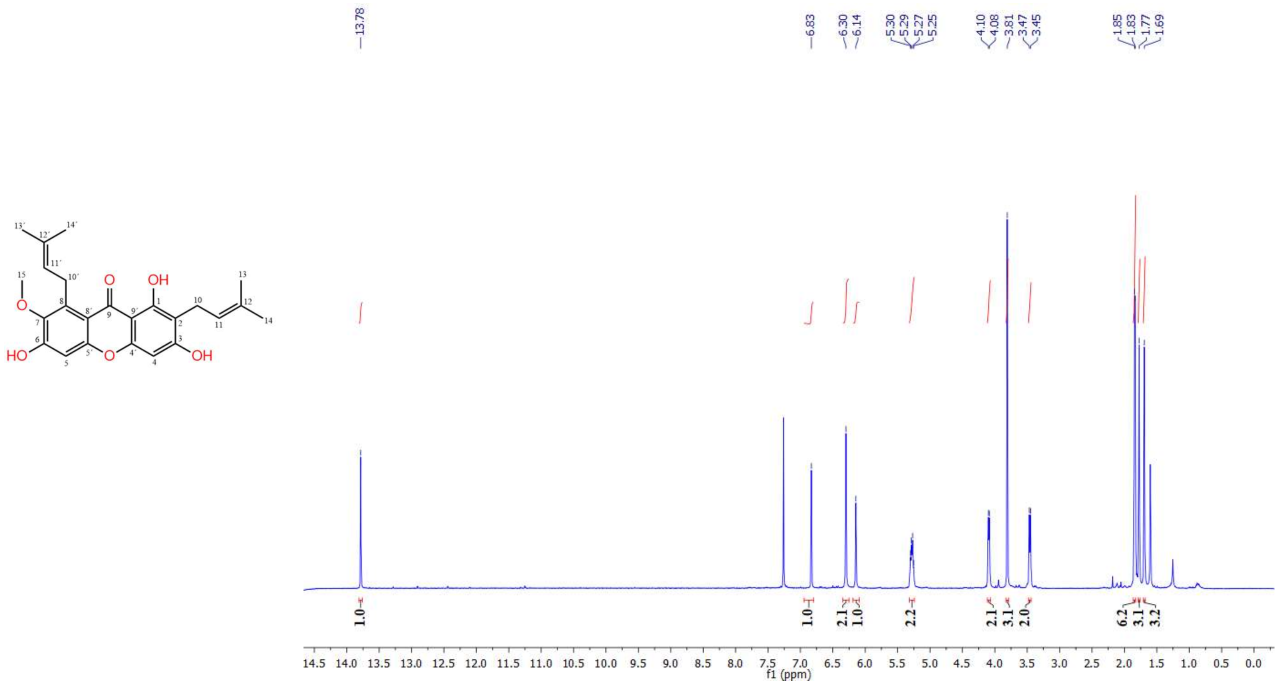

Figures S7. ^{1}H -NMR spectra of *α*-mangostin (**4**).

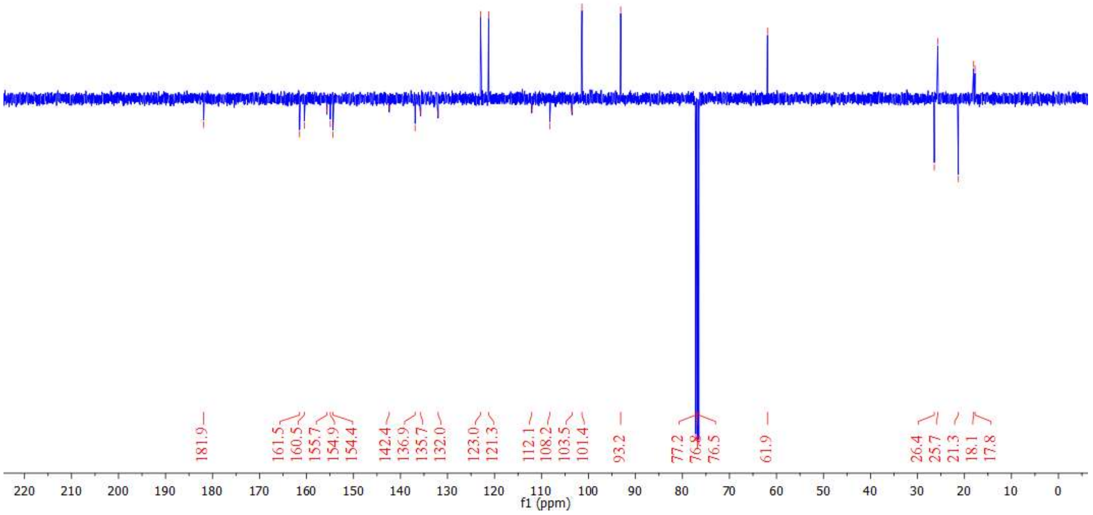

Figures S8. APT spectra of *α*-mangostin (**4**).

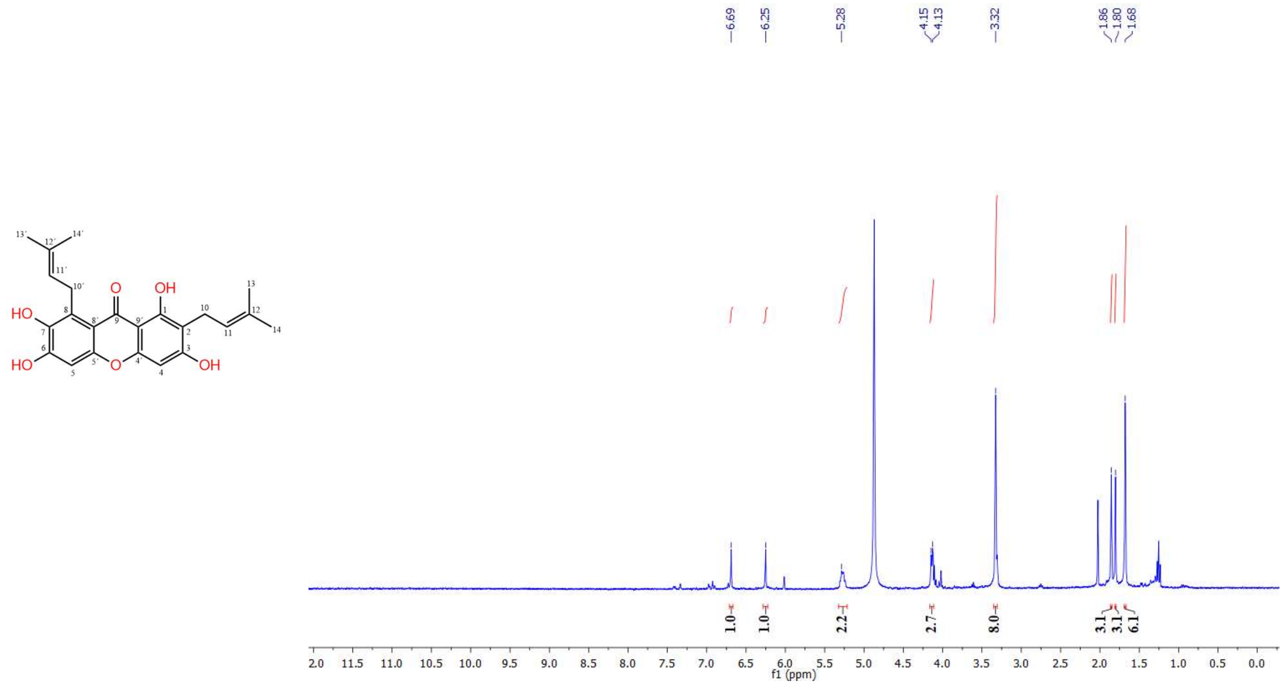

Figures S9. ^{1}H -NMR spectra of γ-mangostin (**5**).

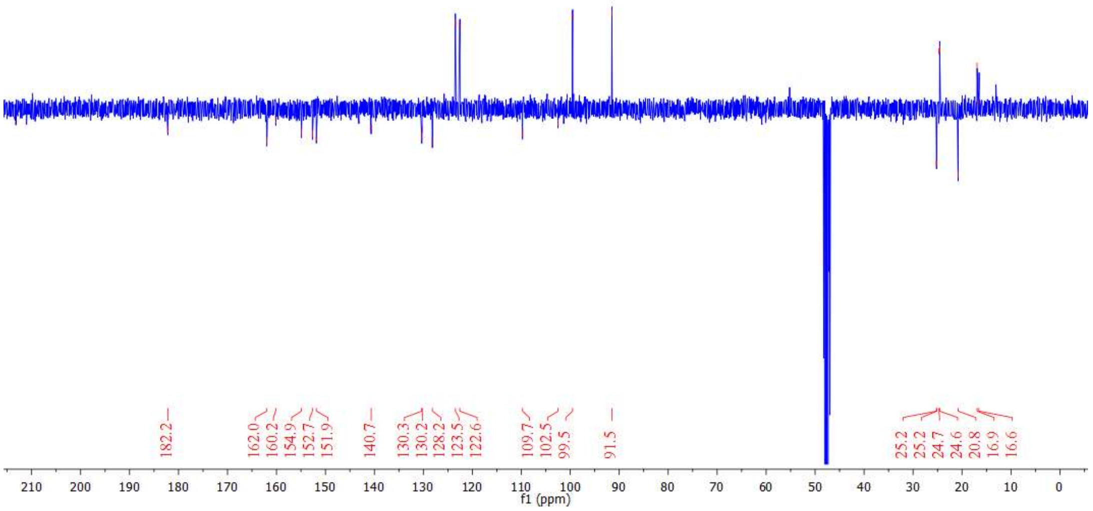

Figures S10. APT spectra of γ-mangostin (**5**).

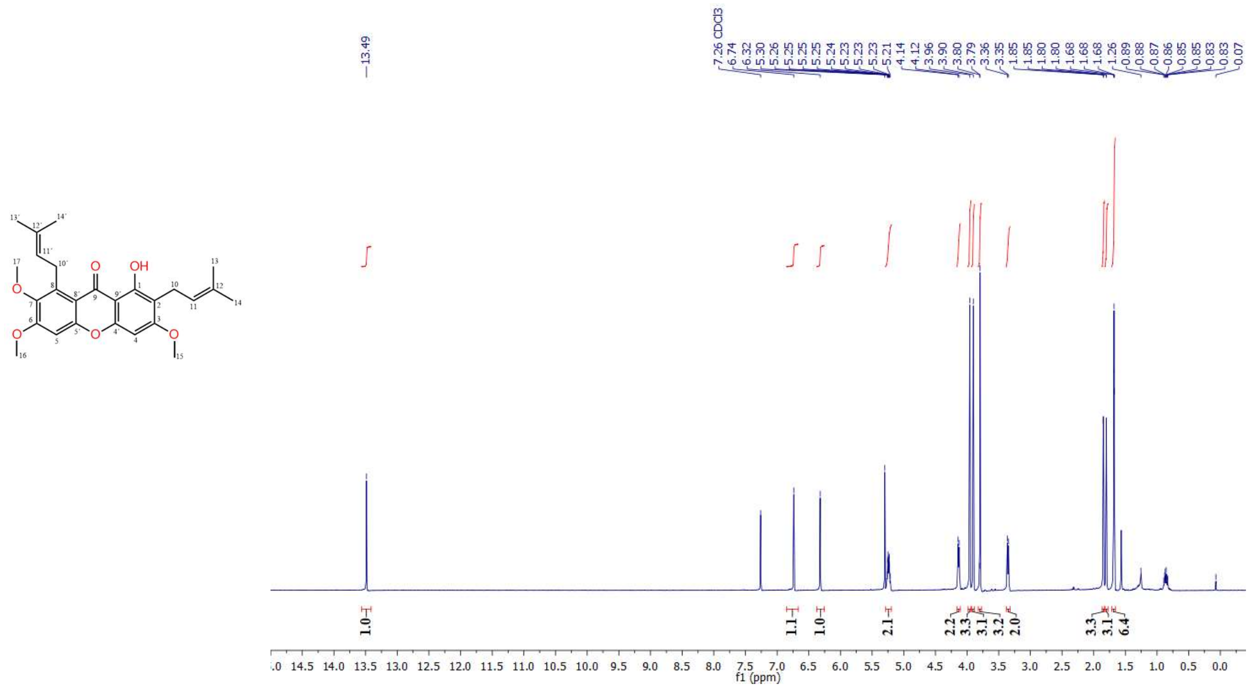

Figures S11. ^{1}H -NMR spectra of fuscaxanthone C (**6**).

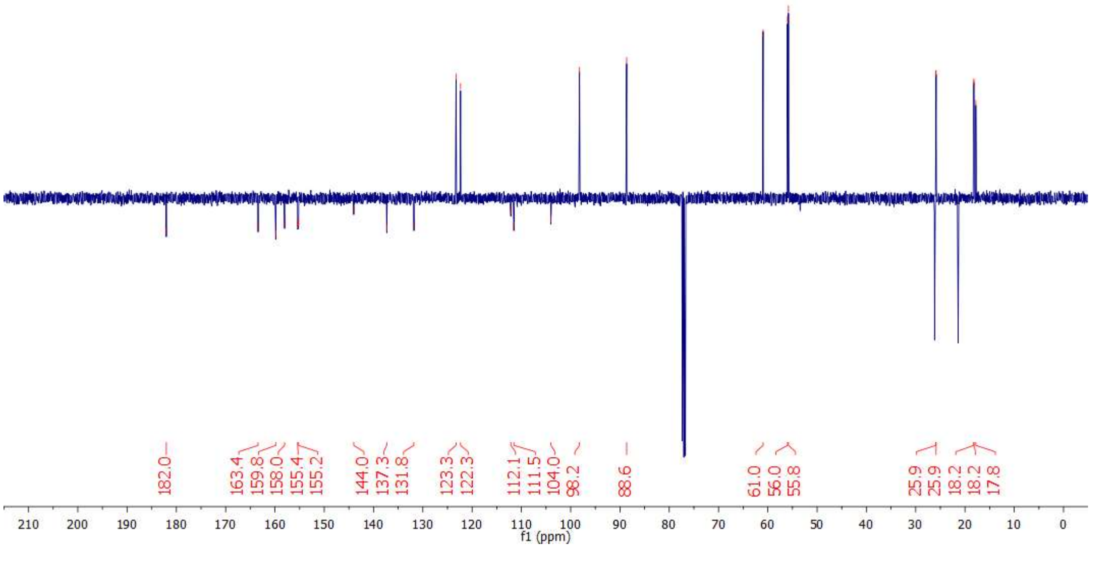

Figures S12. APT spectra of fuscaxanthone C (**6**).

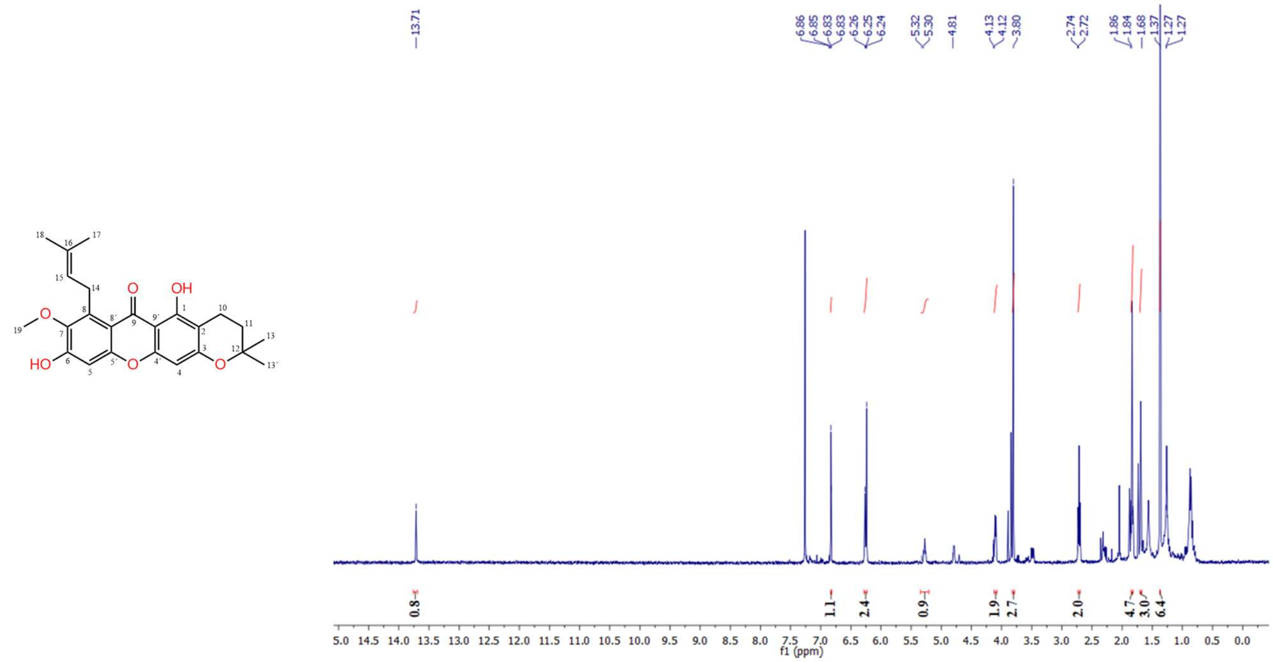

Figures S13. ^{1}H -NMR spectra of 3-isomangostin (**7**).

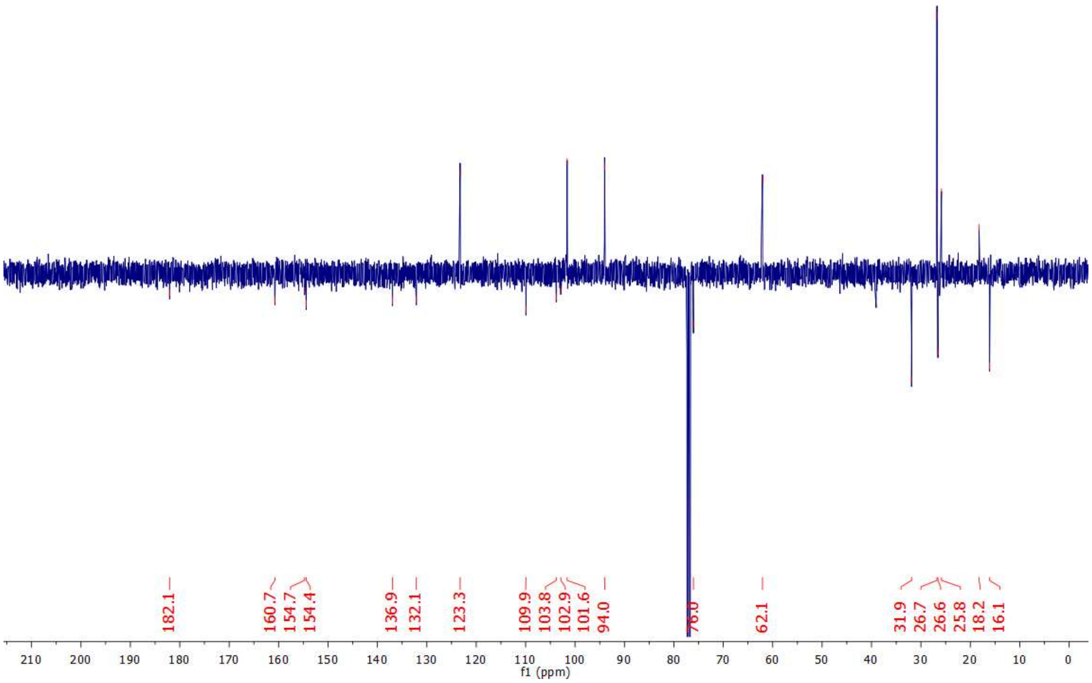

Figures S14. APT spectra of 3-isomangostin (**7**).

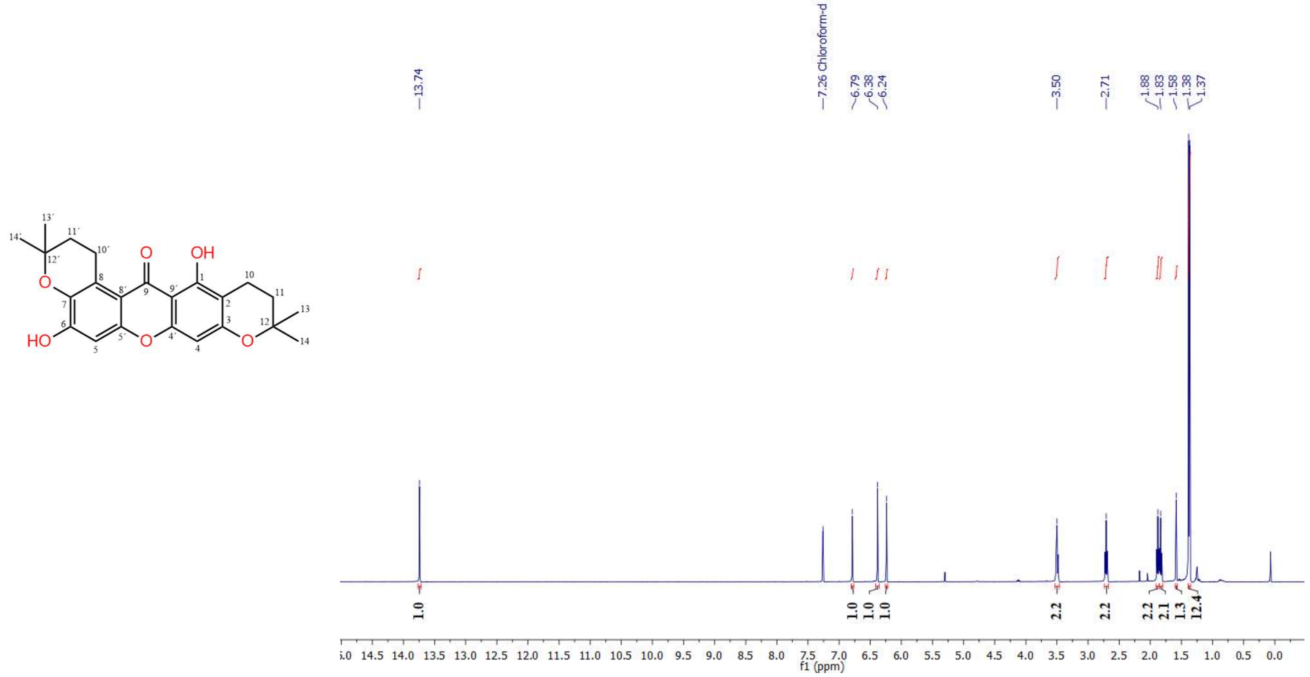

Figures S15. ^{1}H -NMR spectra of BR-xanthone-A (**8**).

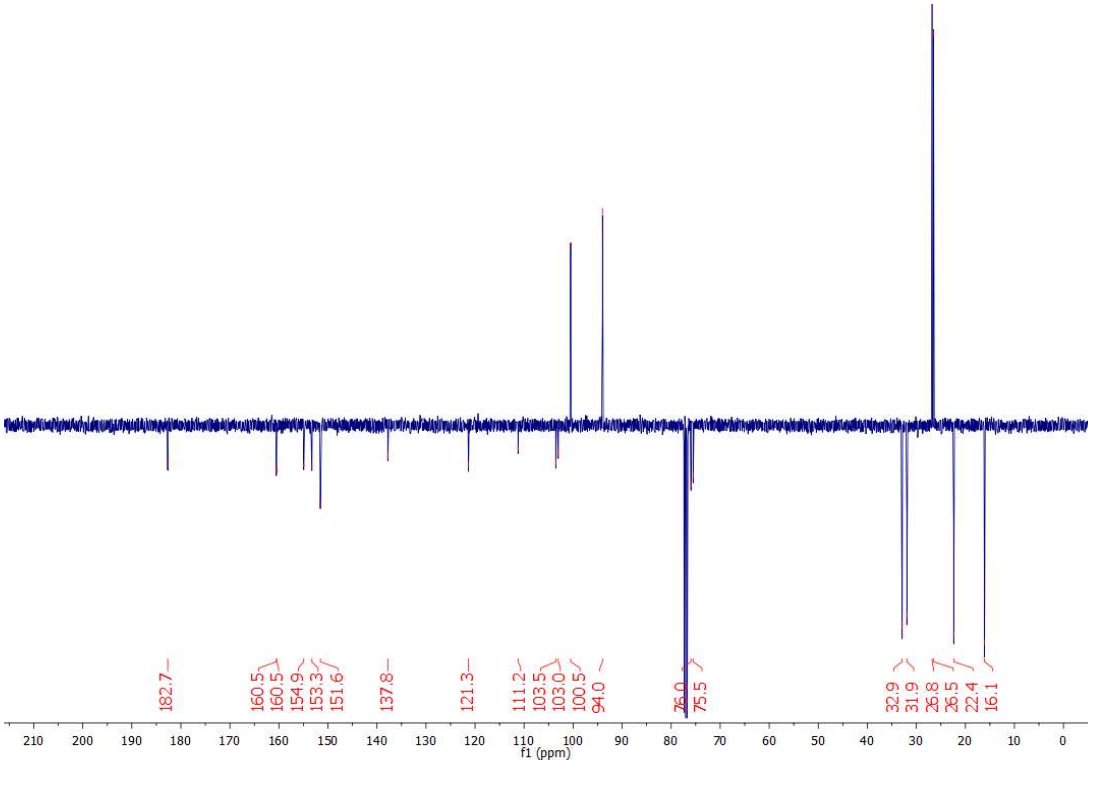

Figures S16. APT spectra of BR-xanthone-A (**8**).

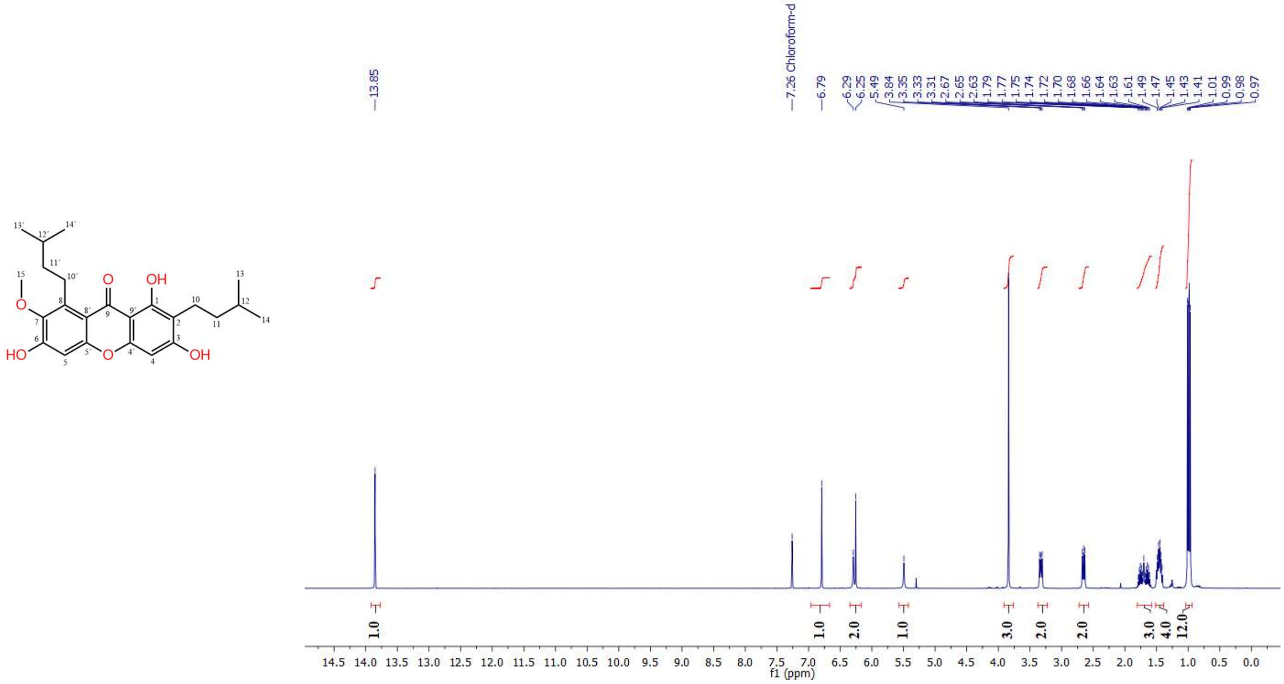

Figures S17. ^{1}H -NMR spectra of tetrahydro-α-mangostin (**9**).

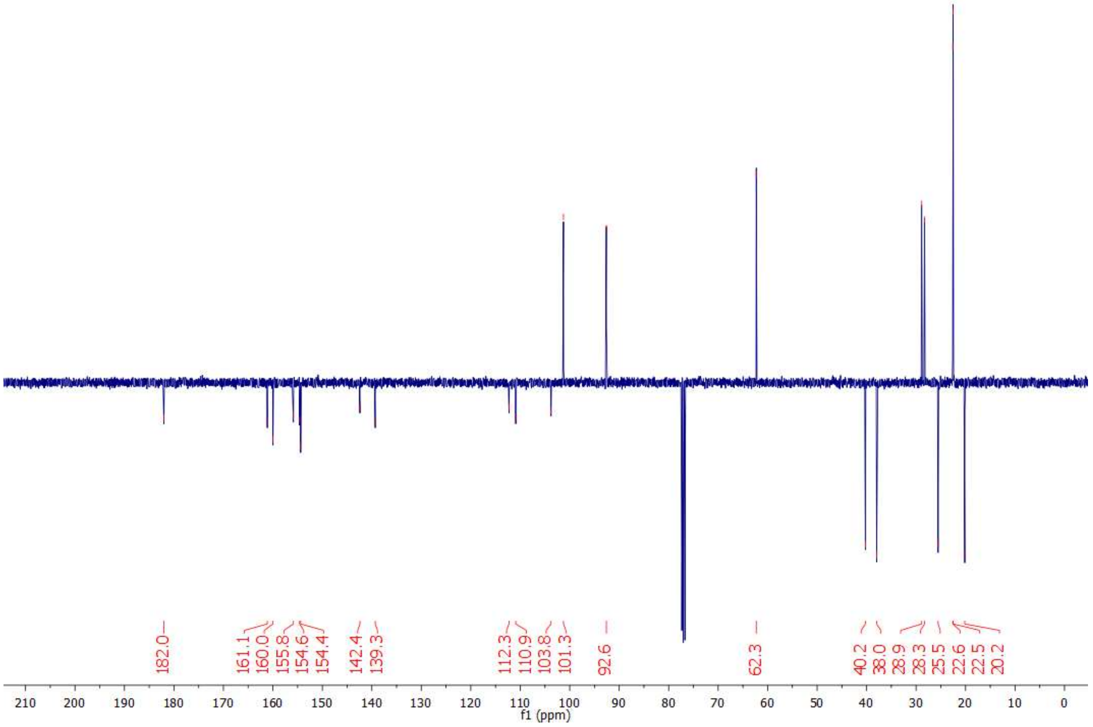

Figures S18. APT spectra of tetrahydro-α-mangostin (**9**).

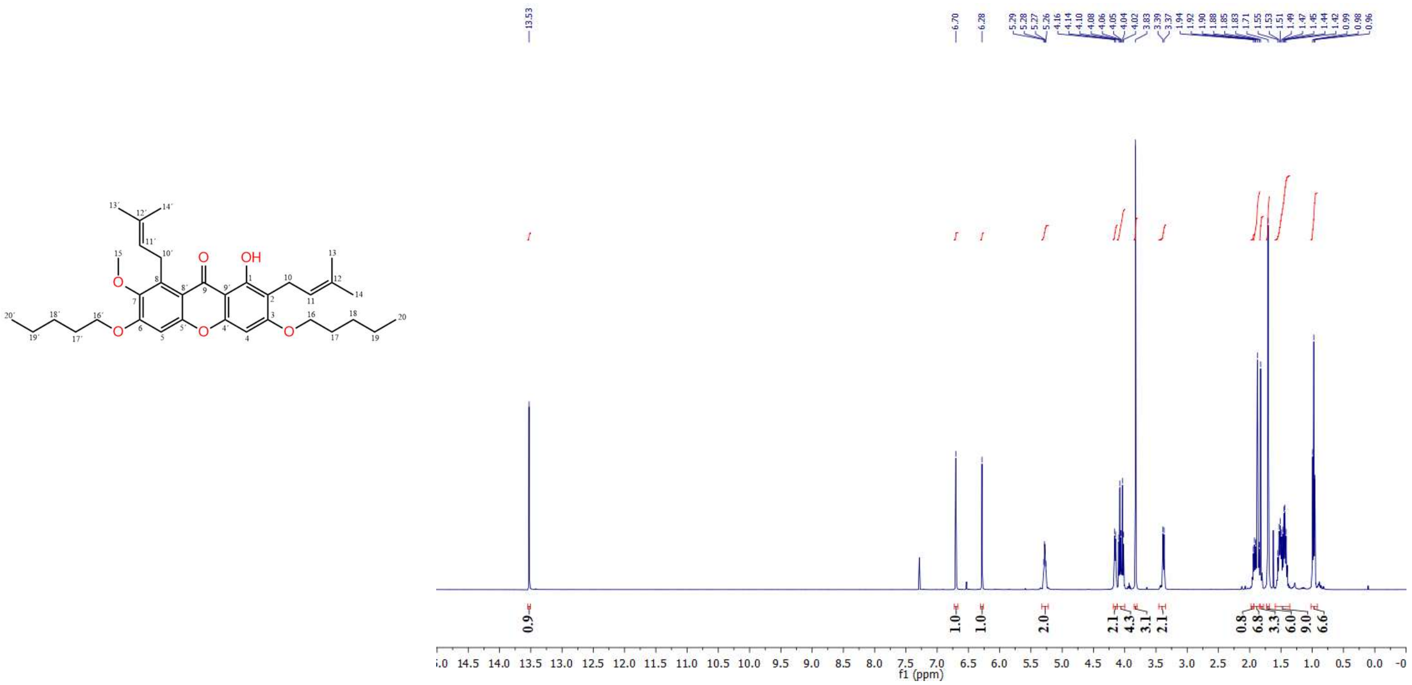

Figures S19. ^{1}H -NMR spectra of 3,6-di-pentoxy-α-mangostin (**10**).

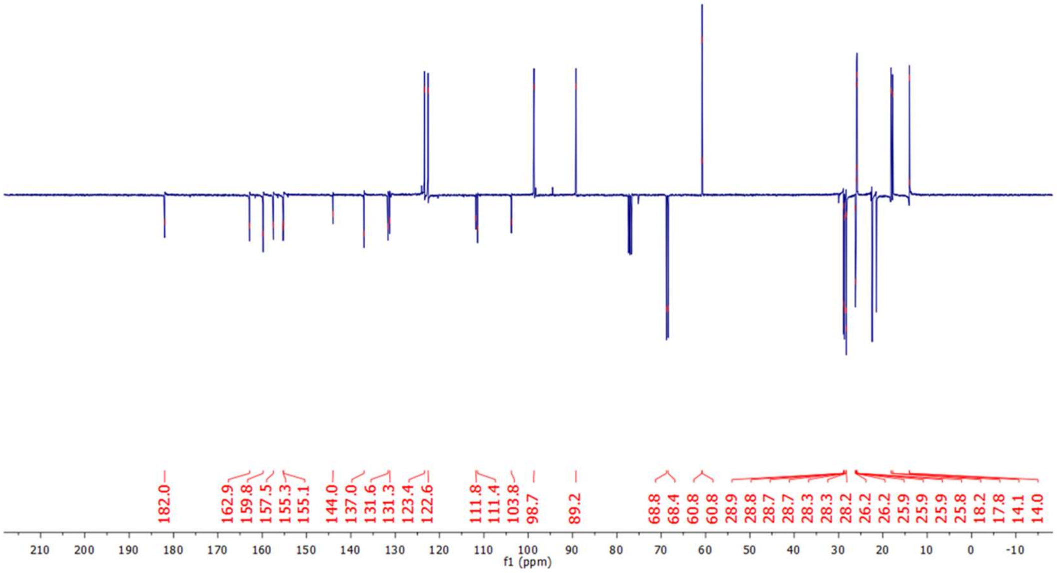

Figures S20. APT spectra of 3,6-di-pentoxy-α-mangostin (**10**).

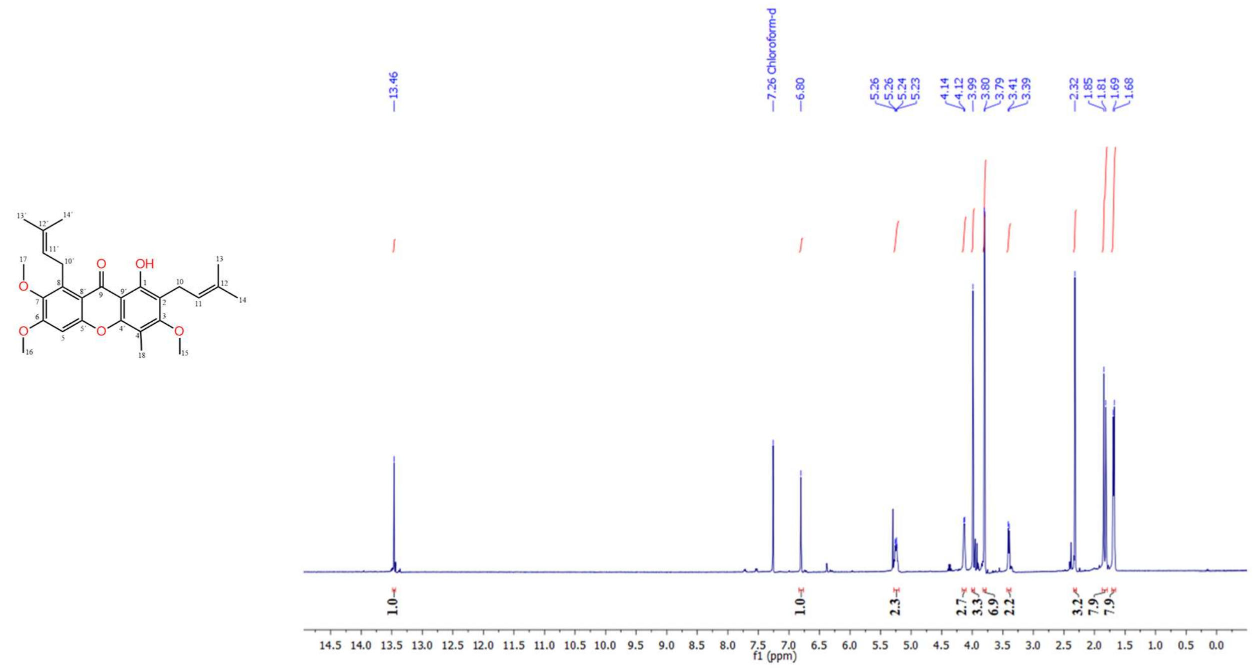

Figures S21. ^{1}H -NMR spectra of 3,6-di-methoxy-4-methyl-α-mangostin (**11**).

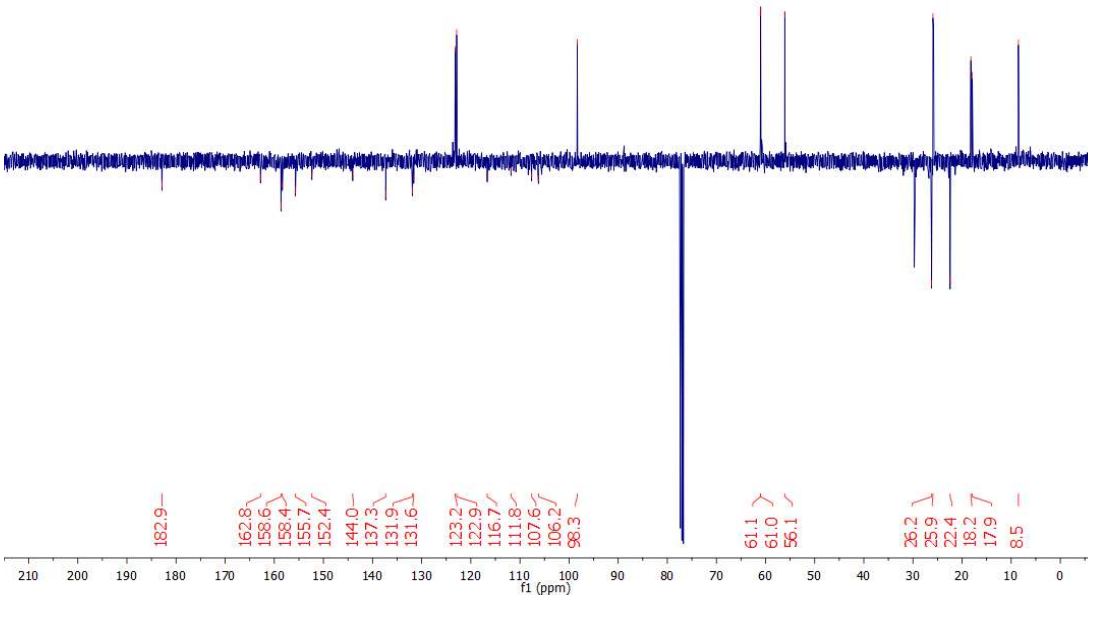

Figures S22. APT spectra of 3,6-di-methoxy-4-methyl-α-mangostin (**11**).

3. Molecular docking studies.

Table S1. Docking Scoring energies found for compounds **1** to **11** against each enzyme (PL, AA and AG)

ADV: AutoDock Vina, ADT: AutoDock 4.2, Glide: Maestro,

	**Pancreatic lipase**			**α-amilase**			**α-glucosidase**		
**Compound**	**Score (Kcal/mol)**								
	**ADV**	**AD4**	**Glide**	**ADV**	**AD4**	**Glide**	**ADV**	**AD4**	**Glide**
**1**	-9,200	-7,080	-6,278				-8,500	-5,560	-3,850
**2**	-8,800	-5,460	-5,782	-7,400	-4,430	-3,554	-7,800	-5,090	-4,603
**3**	-9,400	-4,970	-5,789				-7,800	-4,470	-4,099
**4**	-8,900	-4,740	-7,192	-7,500	-5,030	-3,117	-8,300	-4,280	-3,984
**5**	-9,700	-6,060	-5,801	-8,600	-5,930	-3,089	-8,400	-4,550	-4,160
**6**	-8,900	-4,520	-5,993	-8,100	-3,790	-3,197	-7,500	-4,200	-3,745
**7**							-7,700	-5,180	-3,756
**9**	-9,100	-6,860	-6,894	-7,500	-5,610	-3,476	-8,400	-5,720	-3,394
**11**	-7,200	-8,788	-3,034						

Pancreatic lipase

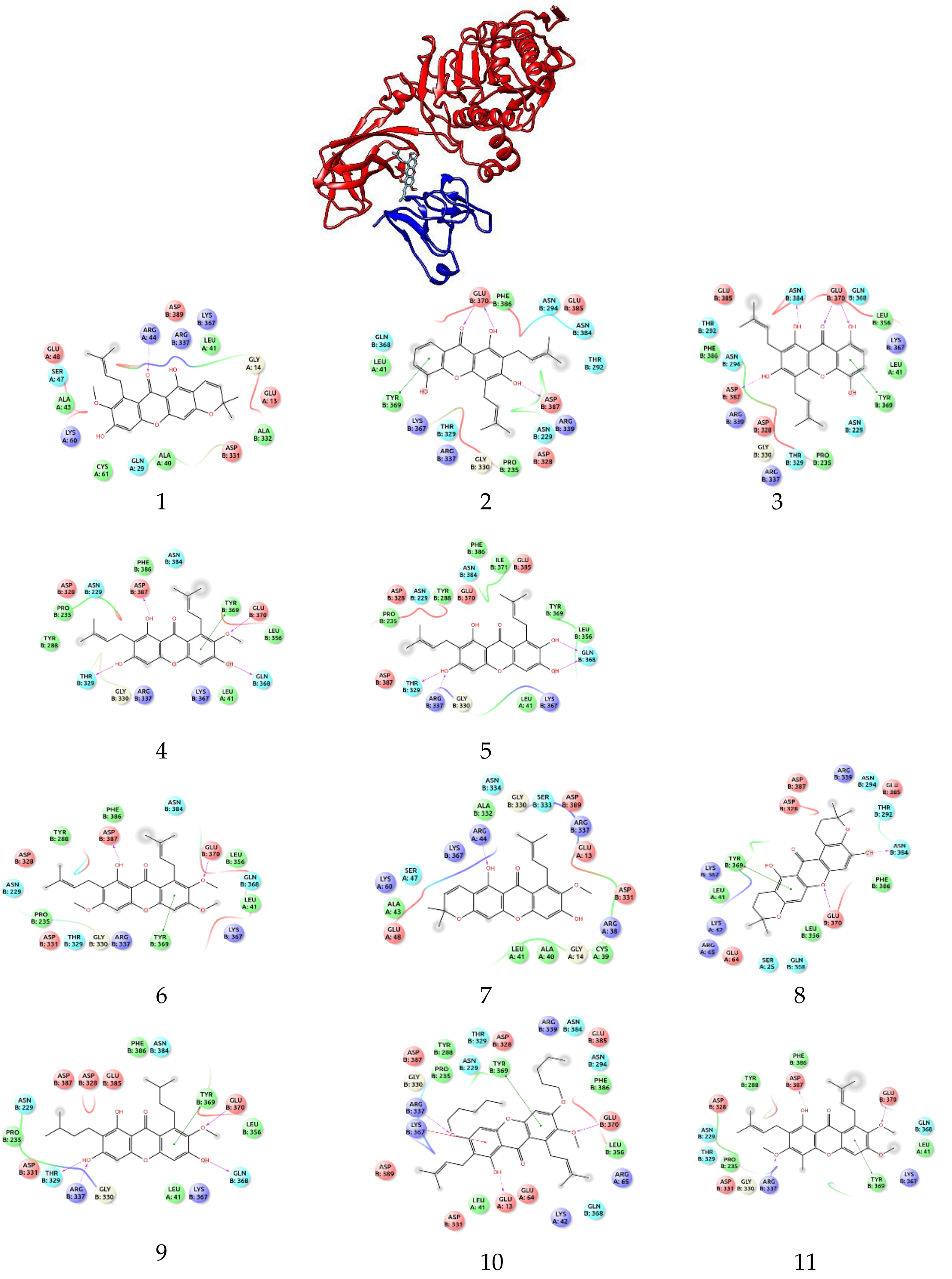

Figure S23. Molecular docking interaction found for compounds **1** to **11** against PL enzyme.

α-amylase

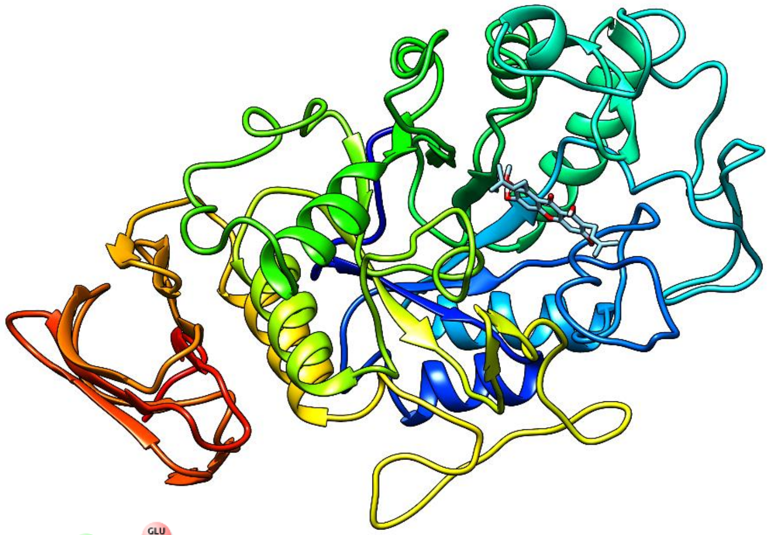

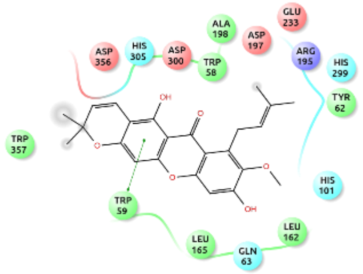

1

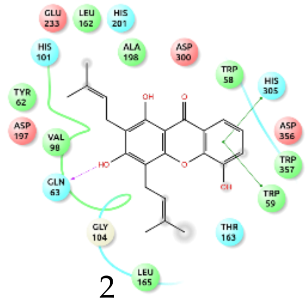

2

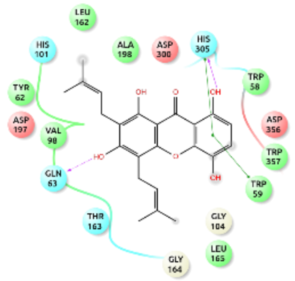

3

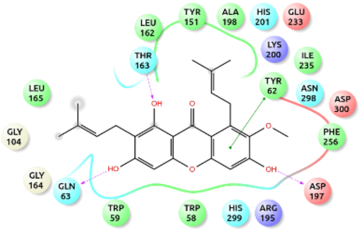

4

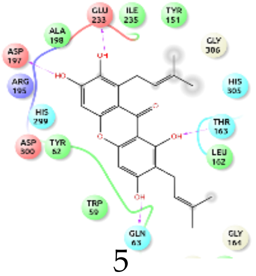

5

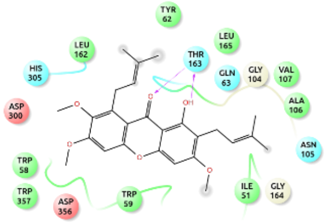

6

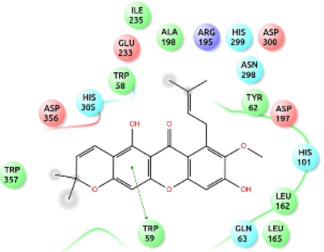

7

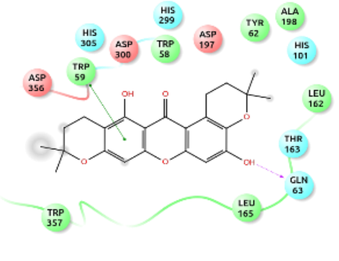

8

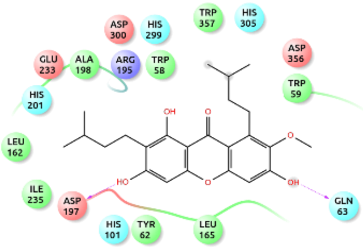

9

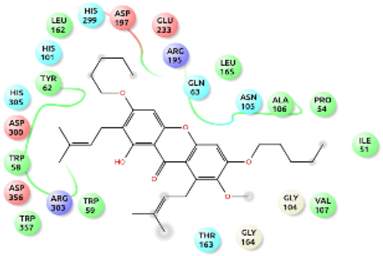

10

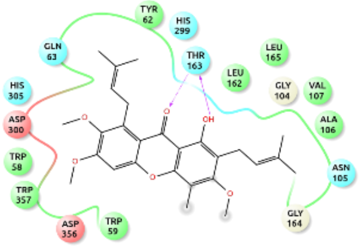

11

Figure S24. Molecular docking interaction found for compounds **1** to **11** against AA enzyme.

α-glucosidase

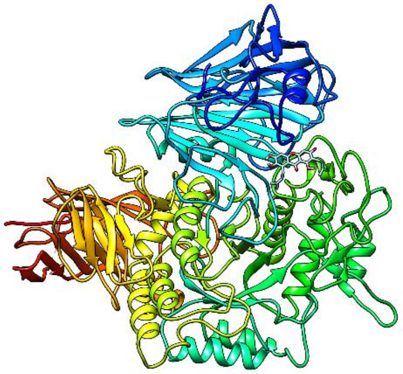

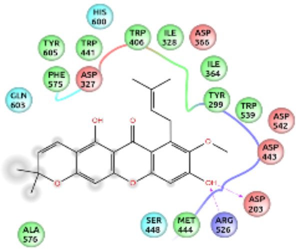

1

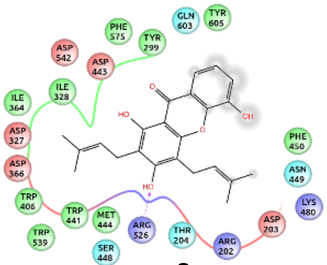

2

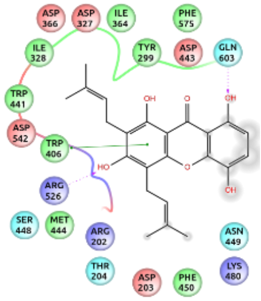

3

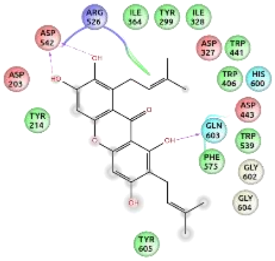

4

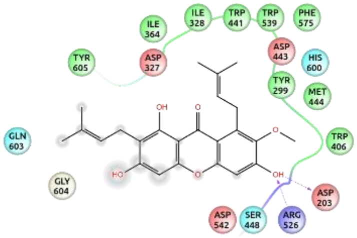

5

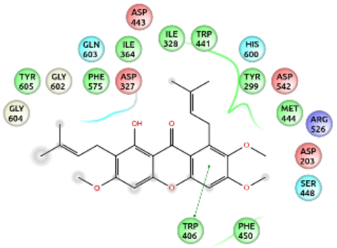

6

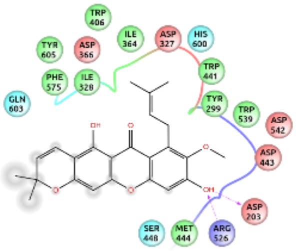

7

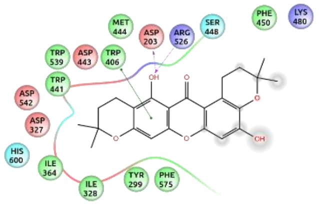

8

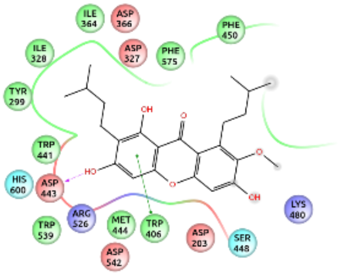

9

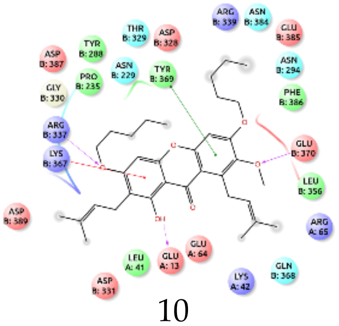

10

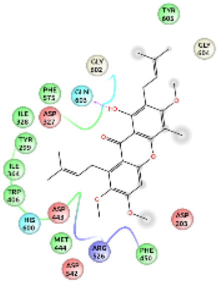

11

Figure S25. Molecular docking interaction found for compounds **1** to **11** against AG enzyme.

4. Kinetic study

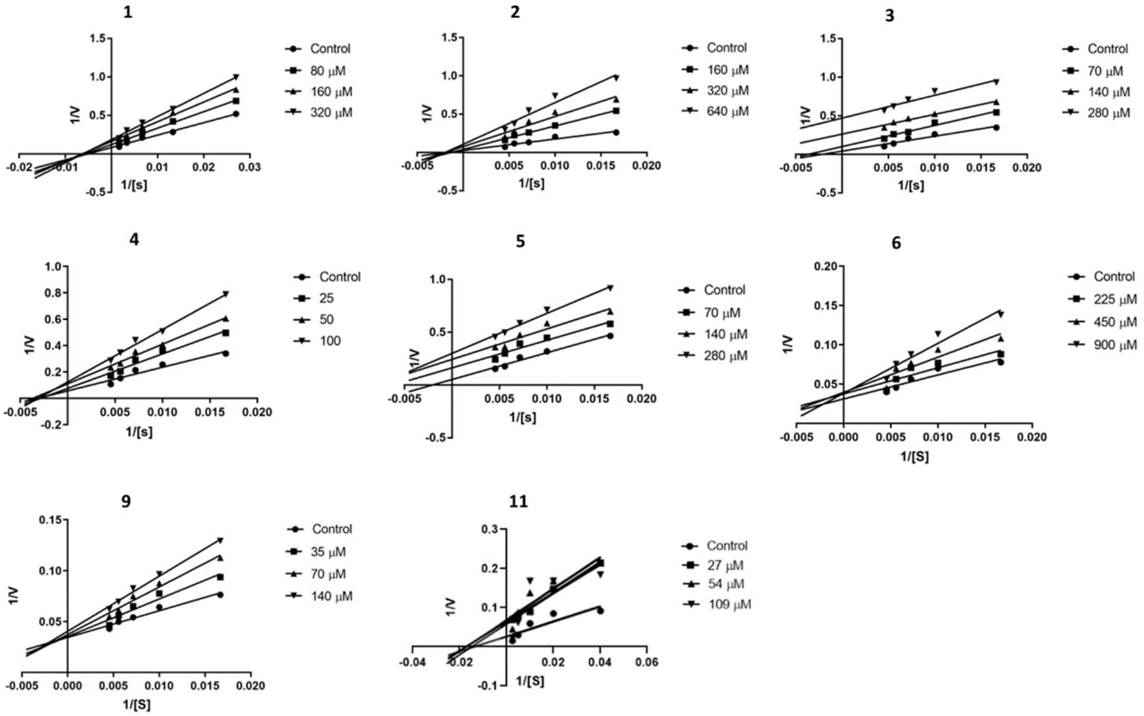

Figure S26. Kinetic found for compounds **1** to **11** against PL enzyme.

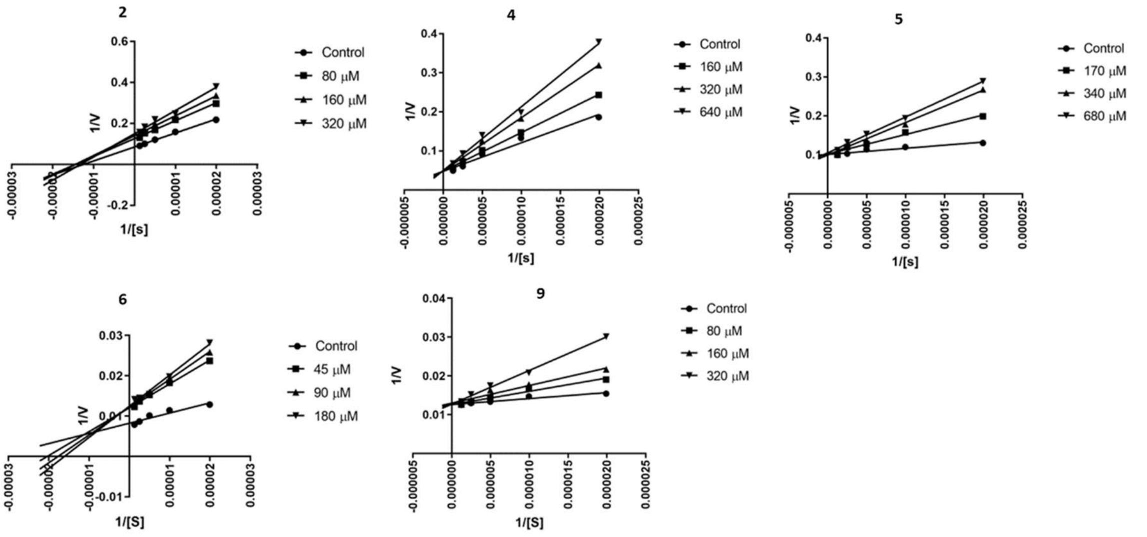

Figure S27. Kinetic found for compounds **2** to **9** against AA enzyme.

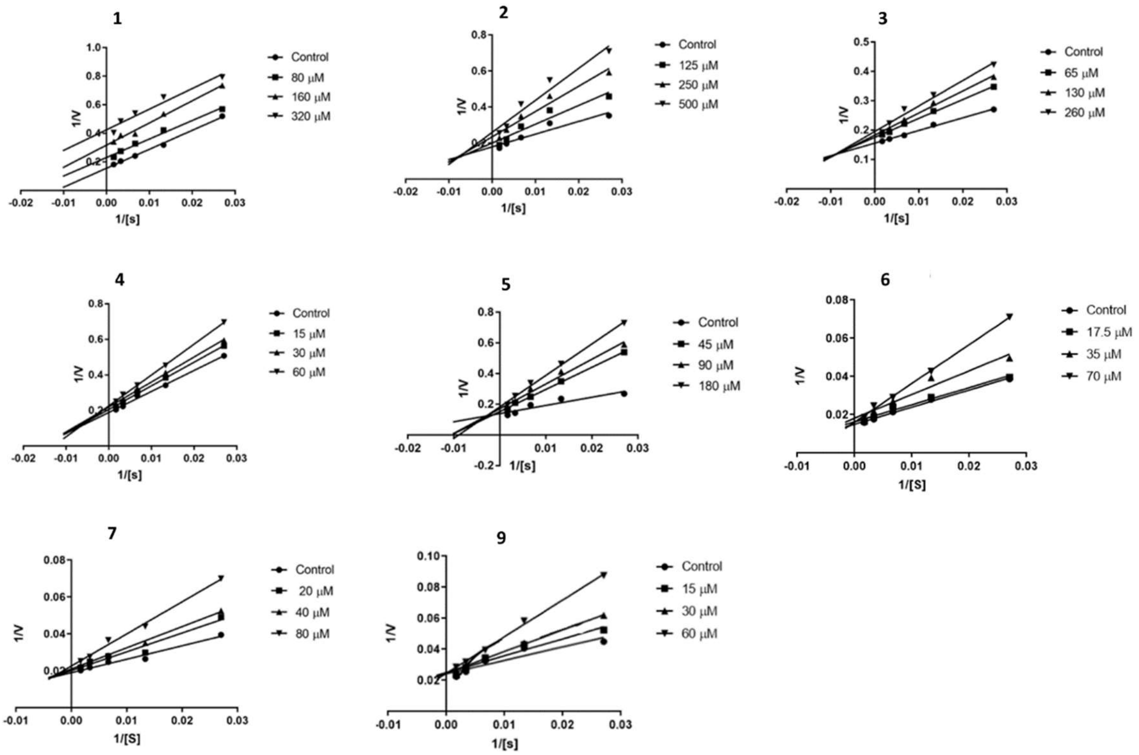

Figure S28. Kinetic found for compounds **1** to **9** against AG enzyme.
